# The Impact of Bariatric Surgery on Cardiovascular Risk Factors and Outcomes: A Systematic Review

**DOI:** 10.7759/cureus.23340

**Published:** 2022-03-20

**Authors:** Mirra Srinivasan, Santhosh Raja Thangaraj, Hadia Arzoun, Siji S Thomas, Lubna Mohammed

**Affiliations:** 1 Internal Medicine, California Institute of Behavioral Neurosciences & Psychology, Fairfield, USA

**Keywords:** premature cardiac death, gastric bypass surgery, quality of life, risk factors, cardiovascular complications, obesity, bariatric surgery

## Abstract

Obesity and its complications are increasing in today's era, with cardiovascular health being one of the most significant obesity-related comorbidities. Hypertension in obesity is considered one of the major causes of death and disability due to their negative repercussions on cardiovascular health. Bariatric surgery is an approved therapeutic modality for obese people in classes II and III who have a body mass index (BMI) of more than 35 kg/m^2^ and 40 kg/m^2^, respectively. These weight loss surgeries are procedures that alter metabolism by causing weight reduction and altering gastrointestinal physiology, thereby considerably decreasing cardiometabolic risk factors that have been poorly understood to date. The purpose of this review is to explore the impact of bariatric surgery on reducing cardiac risk factors, in turn protecting the heart from succumbing to premature death. A literature search was done in the following databases: PubMed, Google Scholar, and PubMed Central (PMC). The studies taken into account for this review were observational studies published between 2016 and 2021 in the English language, where the quality was assessed using relevant quality appraisal methodologies. Finally, 10 reports were selected as definitive studies. Upon extensive evaluation of the final studies, it can be concluded that bariatric surgery results in significant weight loss, which lowers metabolic syndrome prevalence, cardiovascular risk factors, and major adverse cardiovascular events, particularly acute coronary events, and a favorable improvement in cardiac structure and function, altogether steering to reduced mortality due to cardiovascular diseases in obese patients. It is also worth noting that, while metabolic surgery can help patients with various metabolic comorbidities, the impact on individuals with hypertension is still debatable. Although the studies show significant effects on the cardiovascular system, these were only observational studies in geographically dispersed locations where each patient's lifestyle patterns and motivational levels could vary. Since real-world data are not fully explored due to the limited randomized controlled trials, it is suggested that further human trials on a larger scale be conducted to provide an even more factual conclusion.

## Introduction and background

In today's digitalized world, the incidence of obesity has been increasing to a point where negative consequences of obesity are witnessed in adults, especially in the younger generation [1], leading to a shorter lifespan. Obesity (body mass index (BMI) of 30 kg/m² or more) and overweight (BMI of 25 kg/m² or more) are known as abnormal or excessive fat accumulations that pose a health concern [2]. According to the global burden of disease recorded in 2017, four million people die each year due to being overweight or obese [3]. In the United States, obesity prevalence grew from 30.5% to 42.4% between 1999 and 2000. Simultaneously, the prevalence of extreme obesity increased from 4.7% to 9.2% [4]. 

Heart diseases, stroke, type 2 diabetes mellitus (T2DM), and perhaps certain types of cancer are all linked to obesity and are among the major causes of preventable early mortality [4]. Several comprehensive studies have shown an increase in mortality in patients who are above a specific BMI threshold, including impaired glucose tolerance, hypertension (HTN), cardiovascular disease (CVD), dyslipidemia, cerebrovascular disease, metabolic syndrome (MeS), pulmonary abnormalities, and gastrointestinal abnormalities [5].

Cardiovascular diseases are defined as the collection of disorders involving the heart and the blood vessels ranging from coronary artery disease (CAD), cerebrovascular disease, peripheral arterial disease, rheumatic heart disease, congenital heart disease, deep vein thrombosis, to pulmonary embolism. These CVDs are the major cause of mortality worldwide. In 2019, an estimated 17.9 million individuals died from CVDs, accounting for 32% of all global fatalities where myocardial infarction (MI) or a stroke caused 85% of these fatalities [6]. The risk of heart diseases could include an unhealthy diet, physical inactivity, tobacco use, harmful alcohol use, increased blood pressure/blood glucose/blood lipids, overweight, and obesity [6]. The risk of heart failure (HF) was examined across all cardiac diseases in the Framingham research, and the risk of HF was shown to be two-fold higher in the obese group than in the non-obese group [7]. There was a 9% increase in occurrences of ischemic heart disease for every unit change in BMI in the Asian Pacific Cohort Collaboration trial, which monitored over 300,000 people [5]. Due to the trending sedentary lifestyle and rampant increase in unhealthy diet, obesity is considered one of the dreadful risk factors for cardiac diseases that too considered an avoidable cause of death [3].

In order to tackle obesity, there are numerous regimes, including weight loss programs, diet, medications, surgeries, and devices such as electrical stimulation systems, gastric balloon systems, and gastric emptying systems [8]. Do bariatric procedures, when compared to other regimens, result in greater healthspan and restore an individual's overall quality of life in a reasonable amount of time?

Medical therapy that fails to achieve long-term weight loss is typical in people with severe obesity. Intense lifestyle modification can induce an average weight reduction of around 10% after one year and sustain weight loss of 5.3% after eight years [9]. The amount of weight lost varies greatly with each individual as it is a multifactorial approach; however, it has to be significant enough to control comorbidities [10]. According to emerging research, bariatric surgery can result in considerable weight loss and may also aid in the resolution of comorbid diseases, thus enhancing the quality of life [11]. The purpose of this review article is to emphasize the effects obesity may have on the cardiovascular health of an individual and whether bariatric surgery could benefit a patient in preventing cardiac complications, including premature death.

Methodology

The Preferred Reporting Items for Systematic Reviews and Meta-Analysis (PRISMA) guidelines 2020 were followed in this systematic review [12], and the population, intervention, comparison, and outcome (PICO) format was incorporated in this study design.

Inclusion and Exclusion Criteria

The authors' inclusion criteria for this study comprised full-text reports within the past five years (2016-2021) published in the English language across the globe. The study population included human adults, both females and males, suffering from morbid obesity. One side of the group underwent bariatric surgery (intervention), and the other group received conventional treatment for obesity (comparison group) for a desirable overall health outcome. Only cohort and longitudinal studies were included for this research. All other articles before 2016, non-English language, non-full-text articles, and other study designs except those formally mentioned were excluded.

Information Sources, Search Strategy, and Data Extraction Process

A detailed search was done on databases mentioned above using the relevant keywords (disclosed below), and a total of 1,242 articles were identified. Two researchers worked independently to identify and extract data, as well as checked the quality of each study with appropriate quality appraisal tools from November 12, 2021, to November 22, 2021. After removing all duplicates, the reports were evaluated using the authors' inclusion and exclusion criteria, and any irrelevant reports were omitted, including those that did not meet the quality standards. In instances where there were disagreements, both researchers looked at the study designs, inclusion and exclusion criteria, intervention employed, and outcome assessment to come to an agreement. A third author was brought in to resolve differences and find a consensus in ambivalent circumstances. After a complete analysis, 10 reports were finally included in this review.

Keywords

MeSH keywords on PubMed are as follows: Bariatric surgery OR gastric bypass surgery OR weight loss surgery OR ("Bariatric Surgery/adverse effects" [Mesh] OR "Bariatric Surgery/complications" [Mesh] OR "Bariatric Surgery/ethics" [Mesh] OR "Bariatric Surgery/methods"[Mesh] OR "Bariatric Surgery/mortality" [Mesh] OR "Bariatric Surgery/therapy" [Mesh]) AND cardiac risk factors OR cardiovascular diseases OR ("Cardiovascular Diseases/abnormalities "[Mesh] OR "Cardiovascular Diseases/complications "[Mesh] OR "Cardiovascular Diseases/mortality "[Mesh] OR "Cardiovascular Diseases/prevention and control "[Mesh]). Keywords on other databases are as follows: Bariatric surgery; obesity; cardiovascular complications; risk factors; quality of life; gastric bypass surgery; premature cardiac death.

Quality Assessment

**Figure 1:**
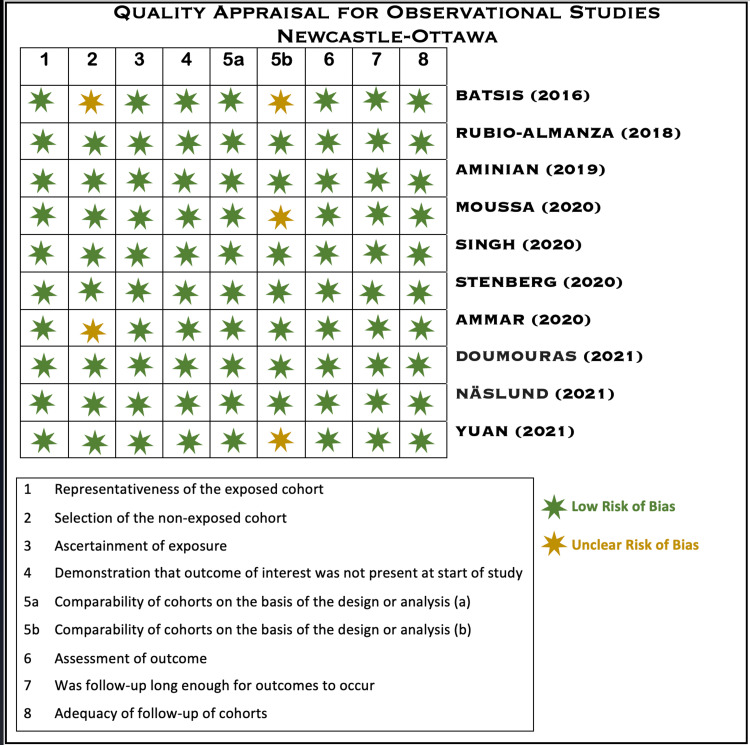
Newcastle-Ottawa Quality Appraisal Tool for Observational Studies

Figure 1 depicts the risk of bias assessed for the included studies by the Newcastle-Ottawa quality appraisal tool.

Results 

**Figure 2:**
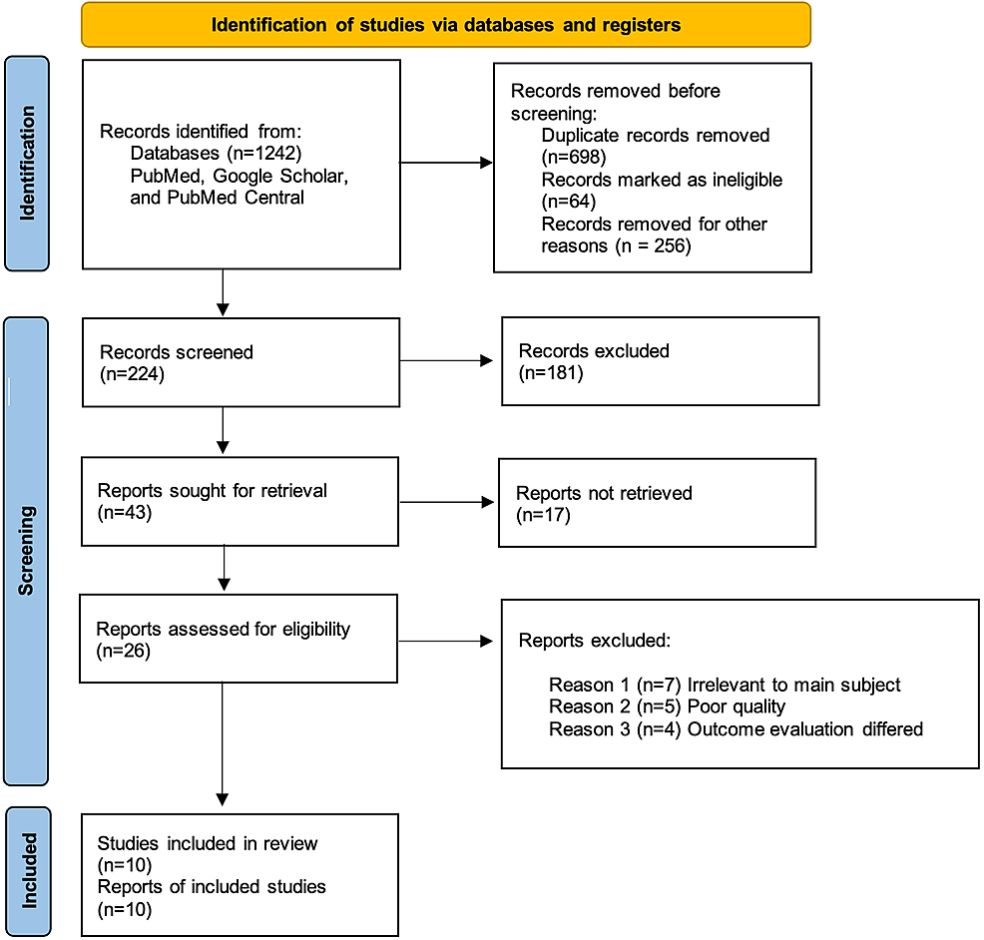
PRISMA Flow Diagram PRISMA: Preferred Reporting Items for Systematic Reviews and Meta-Analysis.

Among the 1,242 articles, 698 were duplicates and were removed using EndNote (Clarivate Analytics, Philadelphia, United States), 64 were removed due to ineligible records, and 256 were removed for other reasons. A total of 224 records were reviewed, with 181 being discarded due to relevancy and inclusion/exclusion criteria, and 17 reports could not be retrieved. The final screening reduced the number of reports to 26, which were evaluated for quality and eligibility. After a thorough reading, the final eligible reports included 10 studies. The study did not use any automation tools. Figure 2 depicts the search process used for this review in the form of a PRISMA flow diagram [12]. 

Table 1 summarizes the conclusion of all included studies in this review. 

**Table 1:** Summary of Results From the Selected Studies RYGB: Roux-en-Y gastric bypass; MeS: metabolic syndrome; T2DM: type 2 diabetes mellitus; CVD: cardiovascular disease; MACE: major adverse cardiac event; HF: heart failure; HTN: hypertension; MI: myocardial Infarct; BMI: body mass index.

Author	Country, Year	Type of Study	Number of Subjects	Type of Bariatric Surgery	Conclusion
Batsis et al. [13]	United States, 2016	Retrospective cohort	40	RYGB	Bariatric surgery causes significant weight loss, reduces cardiovascular risk factors, lowers MeS prevalence, and is an effective treatment in obese elderly patients (>60 years).
Rubio-Almanza et al. [14]	Spain, 2018	Retrospective cohort	105	RYGB	In T2DM and prediabetes, bariatric surgery lowers the risk of CVD.
Aminian et al. [15]	United States, 2019	Retrospective cohort	Exposed=2,287 Control=11,435	Adjustable gastric banding, duodenal switch, RYGB, and sleeve gastrectomy	There was a decreased risk of MACE among patients with T2DM and obesity who underwent metabolic surgery compared to the nonsurgical approach.
Moussa et al. [16]	United Kingdom, 2020	Retrospective cohort	Exposed=3,701 Control=3,701	Not specified	Bariatric surgery lowers the long-term risk of severe cardiovascular events and acute HF in obese individuals.
Singh et al. [17]	United Kingdom, 2020	Retrospective cohort	Exposed=5,170 Control=9,995	Duodenal switch, gastric banding, RYGB, and sleeve gastrectomy	Compared to standard care, bariatric surgery is linked to a lower risk of HTN, HF, and mortality; however, gastric bypass was linked to a lower incidence of CVD compared to standard treatment.
Stenberg et al. [18]	Sweden, 2020	Cohort	Exposed=11,863 Control=26,199	Gastric bypass and sleeve gastrectomy	A lower incidence of significant adverse cardiovascular events, particularly acute coronary events, was associated with metabolic surgery.
Ammar et al. [19]	Egypt, 2020	Longitudinal study	100	RYGB and sleeve gastrectomy	A beneficial improvement in cardiovascular risk profile, cardiac structure, and function was linked to weight loss.
Doumouras et al. [20]	Canada, 2021	Retrospective cohort	Exposed=1,319 Control = 1,319	RYGB and sleeve gastrectomy	In individuals with cardiovascular disease and obesity, bariatric surgery minimized the risk of MACE.
Näslund et al. [21]	Sweden, 2021	Cohort	Exposed=509 Control=509	RYGB and sleeve gastrectomy	Metabolic surgery is significantly associated with a lower incidence of serious complications, MACEs, mortality, new MI, and new-onset heart failure in highly obese individuals who have had a previous MI.
Yuan et al. [22]	United States, 2021	Retrospective cohort	Exposed=308 Control=701	RYGB	Lower incidences of MACE were seen in patients with an early RYGB therapy for BMI reduction.

Focal Points of the Included Studies

Batsis et al. [13]: One year following bariatric surgery, the prevalence of diabetes (57.5% to 22.5%; P<0.03), HTN (87.5% to 73.7%; P=0.003), dyslipidemia (80% to 42.5%; P<0.001), sleep apnea (62.5% to 23.7%; P<0.001), and MeS (80% to 45%; P<0.002) decreased. The baseline risk also decreased from 14.1% to 8.2% at follow-up [13].

Rubio-Almanza et al. [14]: The risk of cardiovascular disease in patients with T2DM and prediabetes was lower than the baseline risk (P=0.001) one year after surgery. BMI, body fat percentage, fasting plasma glucose (FPG), glycated hemoglobin A1C (HbA1c), c-peptide, homeostatic model assessment for insulin resistance (HOMA-IR), low-density lipoprotein cholesterol (LDL-c), systolic blood pressure (SBP), and diastolic blood pressure (DBP) lowered during the first year following the surgery. They had a flat trend or, in some cases, a very slight increase from the 12th to the 60th month. At 60 months, only 3.2% of the patients continued to have a high CVD risk, the remission from T2DM was 92%, and the prediabetic patients did not develop T2DM [14].

Aminian et al. [15]: The primary end-point had six outcomes: the first occurrence of all-cause mortality, coronary artery events, cerebrovascular events, heart failure, nephropathy, and atrial fibrillation. The secondary end-point outcomes included three-component major adverse cardiac event (MACE) (myocardial infarction, ischemic stroke, and mortality) plus the six individual components of the primary end-point. By the end of the study period, 385 patients in the surgical group and 3,243 patients in the nonsurgical group experienced a primary end-point (cumulative incidence at eight years; P<0.001) and an eight-year absolute risk difference [ARD] of 16.9%. All secondary outcomes, including mortality, demonstrated statistically significant differences in favor of metabolic surgery [15].

Moussa et al. [16]: Major adverse cardiovascular events were considerably less common in patients who had undergone bariatric surgery (P=0.001), which was primarily due to a decrease in myocardial infarction (P=0.001) rather than an increase in acute ischemic stroke (P=0.301) in addition to a reduction in new-onset HF (P=0.026) and mortality (P=0.001) [16].

Singh et al. [17]: In terms of combined cardiovascular risk factors, bariatric surgery, on the whole, was not significantly associated with lowering the risk factors; however, Roux-en-Y gastric bypass (RYGB) had a statistically significant association with the combined CVD risk factor (P=0.003). The all-cause mortality (P=0.004), hypertension (P<0.001), and heart failure (P=0.033) were all significantly lower in patients who underwent bariatric surgery regardless of the type of the surgery. These outcomes were similar in patients with T2DM and without T2DM, with the exception of atrial fibrillation, which was reduced in the T2DM group [17]. 

Stenberg et al. [18]: The primary outcome was a MACE, defined as the first acute coronary syndrome (ACS), cerebrovascular incident, fatal cardiovascular event, or unattended sudden cardiac death. MACEs were reported in 379 operated patients (3.2%) and 1,125 control subjects (4.5%). After controlling for hypertension, comorbidities, and education, the metabolic surgery group had a lower risk (P<0.001). When compared to controls, the surgical group had a lower risk of ACS events (P<0.001) and cerebrovascular events (P=0.060). The study's significant deficiencies were the absence of information on BMI and smoking history in the control group besides the nonrandomized study design [18].

Ammar et al. [19]: BMI, heart rate, blood pressure, diabetes, dyslipidemia, and, therefore, metabolic syndrome and Framingham risk score decreased significantly (P=0.0001) six months after surgery. HTN was 24% vs. 12% (P=0.0005), diabetes was 21% vs. 11%, (P=0.002), dyslipidemia was 32% vs. 7% (P<0.0001), and MeS was 54% vs. 26% (P=0.0001). Substantial improvement was seen in all lipid subfractions, and considerable reduction was evident in FPG, two-hour postprandial plasma glucose (2hPPPG), HbA1c, and liver enzymes. Weight loss with bariatric surgery was correlated with a significant reduction in resting heart rate and a shortening of the corrected QT (QTc) interval (P=0.009), which is a predictor of ventricular repolarization. In addition, echocardiographic findings at six-month follow-up revealed a significant reduction in left ventricle (LV) dimensions and LV mass index (LVM) (P<0.0001), a significant increase in left ventricle ejection fraction percentage (LVEF%) (P=0.0003), and an increase in early to late mitral inflow velocity (E/A) ratio (P<0.0001). Age, Framingham risk score, and preoperative BMI had substantial positive correlations with BMI at follow-up [19].

Doumouras et al. [20]: The primary outcome occurred in 11.5% of the surgical group (151/1,319) and 19.6% of the controls (259/1,319) (P<0.001). The association was well defined in individuals with heart failure (P<0.001; absolute risk difference, 19.3%) and ischemic heart disease (P<0.001; absolute risk difference, 7.5%). Surgery also showed a decrease in the risk of the secondary outcome, namely, myocardial infarction, ischemic stroke, all-cause mortality (P=0.001), and cardiovascular mortality (P=0.001) [20].

Näslund et al. [21]: Patients who underwent metabolic surgery had a decreased eight-year cumulative risk of MACEs (18.7% vs. 36.2%), reduced risk of death, and new-onset heart failure, but failed to detect significant changes in stroke or new-onset atrial fibrillation [21].

Yuan et al. [22]: The RYGB within one year (RYGB-1Y) group showed a decreased rate of MACE (P=0.008) and reduced mortality (P=0.04) than the No-RYGB group; however, the RYGB-1Y surgery was not significantly associated with lower atrial fibrillation occurrence (P=0.11) [22].

## Review

In this section, an overview of bariatric surgery including the indications, types, benefits, and risks will be discussed while exploring the mechanisms of after-effects imposed by bariatric surgery on cardiovascular health. A brief note regarding the limitations of this study is also recorded at the end.

Obesity and its complications

**Figure 3:**
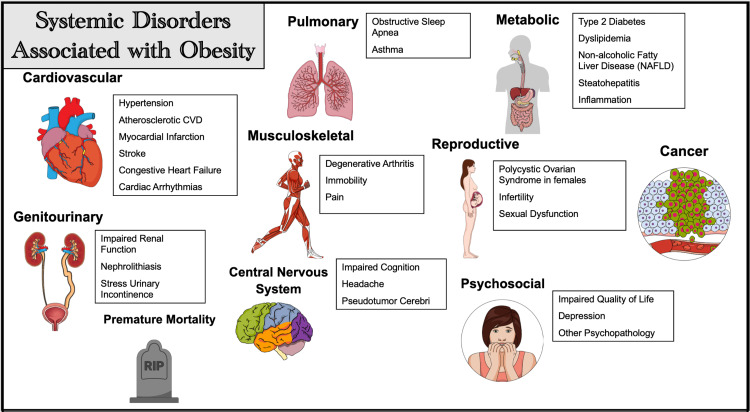
Comorbid Conditions Associated With Obesity Figure created in Mind the Graph platform. CVD: cardiovascular disease.

Comorbidities of obesity are either directly caused by overweight/obesity or are largely attributed to the existence or severity of the disease [23]. Obesity-related complications include heart disease, stroke, T2DM, cancer, digestive issues, gynecological and sexual issues, sleep apnea, and osteoarthritis [24]. It should be emphasized that individuals with abdominal obesity are more prone to develop MeS, T2DM, and CAD; thus, treating obesity is viewed as a crucial step in attaining an overall healthy lifespan [24]. When conservative treatment has not been successful, it is wise to consider more aggressive interventions when assessing the benefits and drawbacks of these procedures [11]. Figure 3 illustrates some of the complications associated with obesity [10].

In the event of sufficient and persistent weight loss, these comorbid disorders are likely to improve or go into remission [10], and multiple studies over the decades have proved that weight loss has the eventual merit to reduce comorbidities, quality of life, and all-cause mortality [10]. With weight loss being the core principle for bariatric surgery in reducing CVD risk [10], the criteria for bariatric surgery are constantly evolving, taking into account the presence or absence of comorbid conditions and the severity of the obesity, as reflected by BMI [25]. Given the safety and effectiveness of bariatric surgery, it is underused, with less than 1% of adults with obesity considering it. Furthermore, with the increasing trend of obesity worldwide, more people should have access to bariatric surgery [26].

An overview of bariatric surgery

Any gastric bypass surgery done in an effort to reduce weight is collectively known as bariatric surgery [27]. Metabolic and bariatric surgeries have been optimized for decades and are among the most extensively researched treatments in modern medicine [28], where techniques using minor incisions through laparoscopic approaches and even robotic surgeries are performed [27]. A National Institute of Health consensus panel defined a surgical strategy to extreme obesity in 1991 [29], where a criterion was established to treat this disease, which has multiple adverse effects on health that can be reversed and improved by significant weight loss in patients who were unable to attain weight loss through nonsurgical means [10]. The indications for the bariatric surgery are included as follows: when less invasive weight loss techniques have been unsuccessful, and the patient is at high risk for obesity-related morbidity or mortality, surgery is an option for selected patients with clinically severe obesity (BMI of 40 kg/m² with concomitant diseases) [11]. Other indications include a viable weight-loss option for well-informed and motivated individuals with a BMI of 35-40 kg/m², concomitant diseases, and tolerable surgical risks [11]. The availability of a multidisciplinary team that could monitor the patient after the surgery with lifelong medical surveillance is also essential [11]. These criteria have continued to remain the same for almost 30 years, albeit specific indications for bariatric/metabolic surgical intervention have been identified for those with less extreme obesity, such as people with a BMI of 30-35 kg/m² who also have T2DM [25].

Types of bariatric surgery

**Figure 4:**
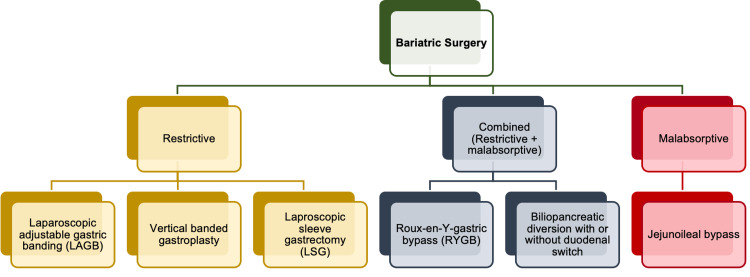
Classification of Bariatric Surgery Figure created by the author on Microsoft Powerpoint.

Bariatric surgical procedures can be classified into three cardinal types depending on the function: restrictive, restrictive plus malabsorptive (combined), and malabsorptive, where all three types are regarded as permanent [11]. Figure 4 summarizes the different subtypes of gastric bypass surgery [11].

**Figure 5:**
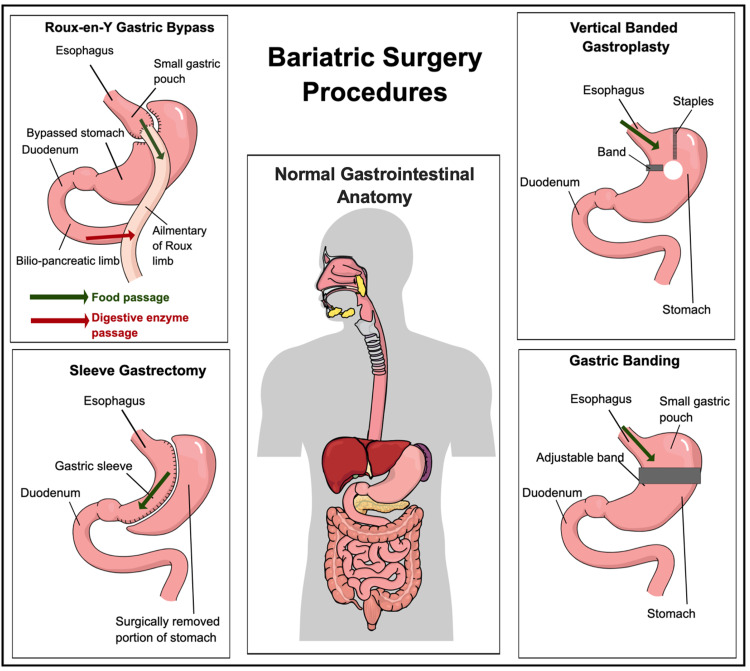
Bariatric Surgery Procedures Figure created in Mind the Graph platform.

Figure 5 illustrates a brief understanding of each operating gastric bypass procedure [30,31].

The sleeve gastrectomy and gastric bypass are the two most common procedures done today and seem to have similar results on weight reduction and diabetes outcomes, as well as have identical safety profiles after a five-year follow-up. However, new research reveals that the sleeve treatment is linked to fewer reoperations, while the bypass procedure may result in longer-lasting weight loss and glycemic management [32]. The study by Athyros et al. concluded that in terms of weight loss and comorbidity resolution, laparoscopic Roux-en-Y gastric bypass (LRYGB) appears to be more effective than laparoscopic adjustable gastric banding [33]. Although safety is a priority, recent data show that perioperative death rates range from 0.03% to 0.2% [32], indicating that they have improved significantly since the early 2000s. Regardless of the type of surgery, actual data suggest that surgery leads to more significant weight loss and T2DM outcomes when compared to non-surgical approaches [32]. Could research by Arterburn et al. [32] possibly open gates to aid in replicating the benefits of bypass surgery in reducing the cardiovascular complications of obesity?

Benefits and complications of bariatric surgery

**Figure 6:**
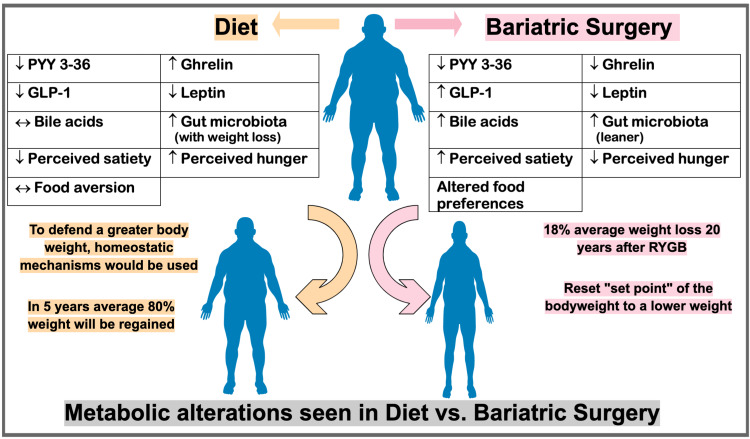
Metabolic Alterations in Diet vs. Bariatric Surgery Figure created in Mind the Graph platform. GLP-1: glucagon-like peptide- 1; PPY: peptide YY; RYGB: Roux-en-Y gastric bypass.

The benefits include a multisystem effect that provides for a reduction in blood pressure, blood glucose levels, blood cholesterol, and triglyceride levels; increase in high-density lipoprotein levels; improvement in liver steatosis, inflammation, and fibrosis; resolution of non-alcoholic fatty liver; decrease in albuminuria and glomerular hyperfiltration; reduction in left ventricular mass index; resolution of obstructive sleep apnea; and reduction in CAD [33]. Patients not only lose weight and keep it off, but there is now compelling evidence that many of them are curable of obesity-related diseases, the most common of which is T2DM [34]. Weight loss through calorie restriction increases ghrelin levels, resulting in an insufficient long-term efficacy of dietary manipulation in preventing obesity; however, in gastric bypass surgeries, the ghrelin levels are reduced due to curtailed interaction between ghrelin-producing mucosal cells and ingested food [35]. Best of all, morbidly obese patients who undergo this surgery appear to have a reduced long-term mortality risk than those who do not [34]. Figure 6 emphasizes the difference encountered in one's metabolism when treated with a controlled diet versus bariatric surgery in obese individuals [30].

**Figure 7:**
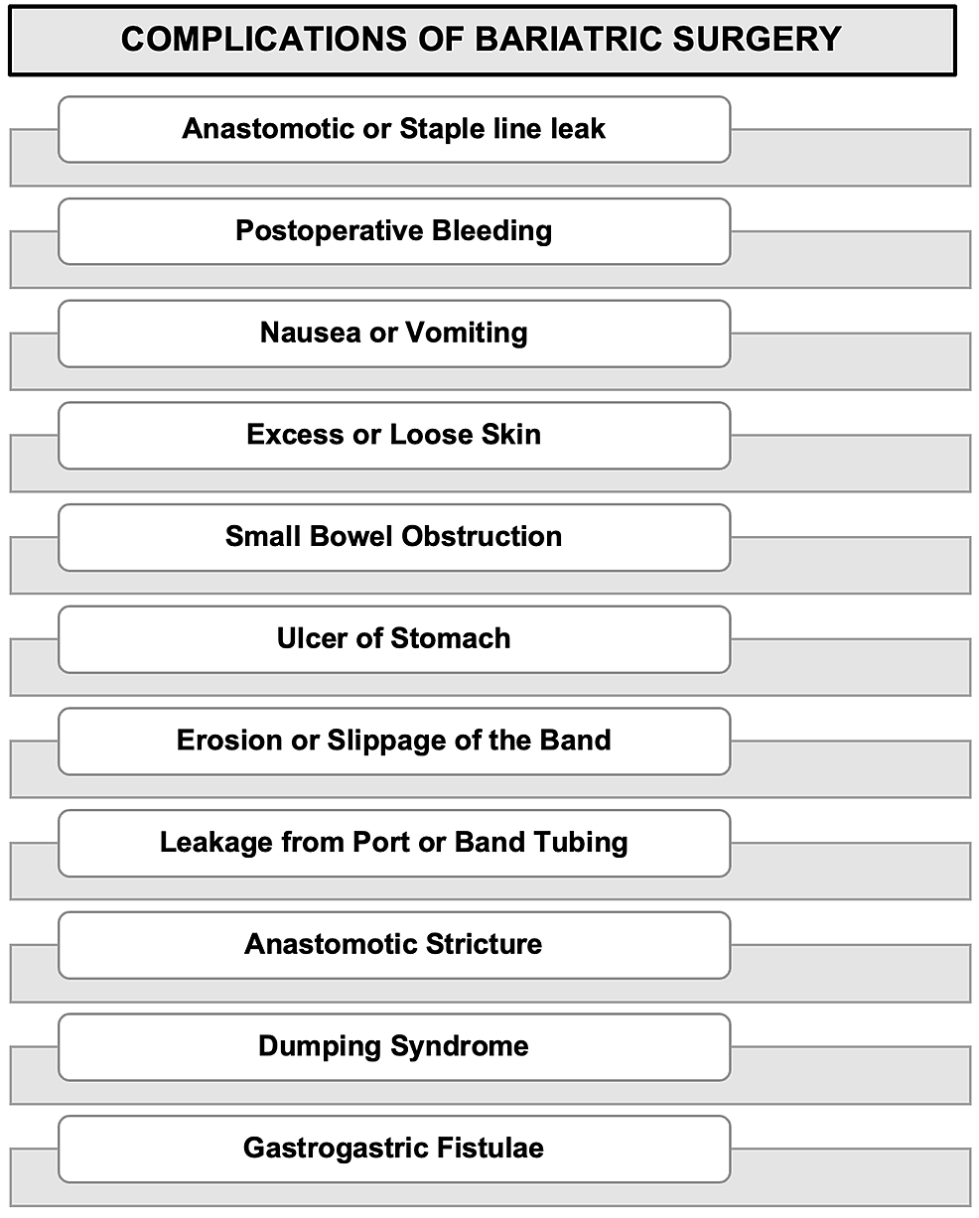
Possible Complications of Bariatric Surgery Figure created by the author on Microsoft Powerpoint.

Patients with BMIs less than 50 kg/m² and younger than 55 years of age have a mortality rate of less than 1%, while patients with a BMI of more than 60 kg/m² with concomitant illnesses have a mortality rate of 2% to 4% [11]. Surgical volume at the clinic (>100 cases per year), surgeon's experience, surgery in a tertiary care center, patient's sex (female), patient's age (55 years), and respiratory state all have a favorable correlation with lower risk of morbidity and mortality [11]. The average complication rate for bariatric surgery is less than 10% [36]. Figure 7 depicts the possible complications of bariatric surgery regardless of the type of surgery [37].

Effects of bariatric surgery on the cardiovascular health

**Figure 8:**
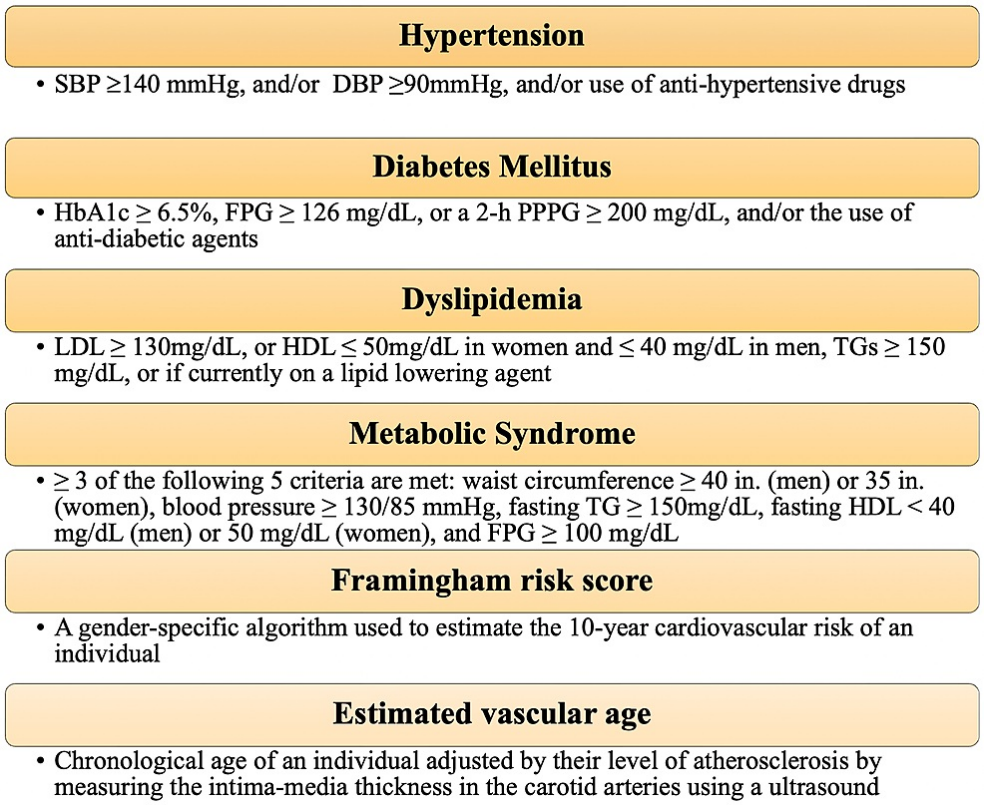
Cardiovascular Risk Factors in Obesity Figure created by the author on Microsoft Powerpoint. SBP: systolic blood pressure; DBP: diastolic blood pressure; HbA1c: glycosylated hemoglobin A1C; FPG: fasting plasma glucose; 2-hPPPG: two-hour postprandial plasma glucose; LDL: low-density lipoprotein; HDL: high-density lipoprotein; TG: triglycerides.

Figure 8 enumerates the typical cardiovascular risk factors seen in obesity [19].

**Figure 9:**
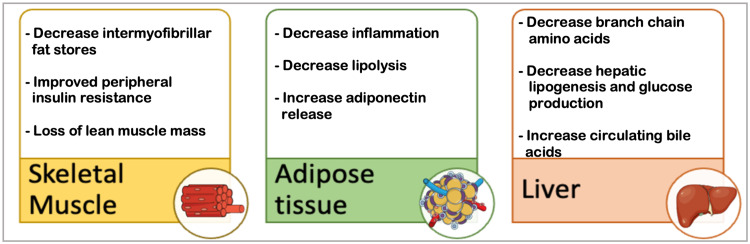
Metabolic Consequences Following Bariatric Surgery Figure created in Mind the Graph platform.

Following bariatric surgery, the body undergoes a multilevel metabolic alteration which in turn impacts the cardiovascular system [38]. A complete mechanism of these variabilities is not fully understood [39]; however, numerous researches have postulated answers to this concern. One study states that this alteration primarily begins with an early transfer of nutrients, modifications in nutrient absorption in the gut, change in bile flow, and variations in the microbiota profile [40]. These alterations in turn release incretin and the satiety hormone in the stomach while decreasing food intake, satiations, and fat preference in the brain. The pancreas reacts by reducing steatosis and insulin secretion while increasing the beta-cell function [41]. The resultant metabolic derangement impacts the skeletal muscle, adipose tissue, and the hepatic system in the following ways, as illustrated in Figure 9 [40].

A decrease in testosterone and estradiol levels occurs, ultimately increasing the sex hormone-binding globulin [40]. In terms of the kidney, there will be an increase in the fractional excretion of sodium, and thus, improved markers of renal damage and inflammation can be evident [40]. In the liver, a fibrotic and inflammatory deterioration along with decreased steatosis will occur in addition to a decrease in bone mineral density, and a smaller amount of bone loss can be noticed [41]. One study postulated a timeline of sequential mechanisms in weight loss after the RYGB procedure, starting with energy restriction, leading to a decrease in hepatic glucose production in the early days. After a few weeks, postoperatively, rapid nutrient passage to the small intestine will occur, increasing glucagon-like peptide 1 (GLP-1), peptide YY (PYY), and oxyntomodulin (OXM). This increases postprandial insulin secretion and decreases postprandial plasma glucose [42], eventually leading to increased satiety and weight loss that is bound to occur as months pass by. Weight loss enhances peripheral insulin sensitivity in general, leading to improved glycemic management [42], and, as a result, protects the heart by reducing risk factors.

Changes in heart function and structure, both systolic and diastolic, have been observed following bariatric surgery [19]. In the study by Cuspidi et al., bariatric surgery demonstrated to induce the following in obese patients with preserved LV function: (1) notable depletion of absolute LVM where the LVM was indexed to body surface area or height, and relative wall thickness (RWT) and all established indexes of LVH and LV geometry have shown to anticipate cardiovascular outcomes; (2) an increase in mitral flow E/A ratio was seen which improved LV diastolic function; (3) decrease in left atrial size, indirectly indicating a normalizing LV filling pressure and diastolic function; and (4) no remarkable changes in LVEF [43].

Furthermore, it can be said from other observational studies that metabolic surgery may be a beneficial secondary prevention strategy in obese individuals with already established coronary artery disease [21]. This implies that preexisting cardiac disorders do not have any contraindication for undergoing bariatric surgery since the rate of complications following bariatric surgery in both groups (previous MI and no previous MI) was the same [21].

Limitations

This study did not detail the risks and benefits of each kind of gastric bypass surgery. The focus was more on the postsurgical benefits, assuming that the mortality and morbidity due to the procedure itself would be uneventful. The reports selected emphasized a retrospective analysis, thus leading to few constraints the study design itself could have, for example, the motivation levels of individuals may have varied, patients could have been lost in the follow-up, etc. This study only explored the cardiometabolic risk factors, including cardiac structure and function. On the contrary, it did not probe too much into cardiac rhythm disorders such as atrial fibrillation, which could be a separate domain by itself to be researched.

## Conclusions

Metabolic surgery may not be the perfect solution for obesity. However, because high blood pressure and obesity are two of the most common causes of mortality and morbidity around the world, metabolic surgery can be considered a reasonable aspect of treatment if it is feasible. After a complete understanding of the cardiovascular risk factors seen in obese patients, it can be concluded that the normalization of metabolic parameters starting with lipid levels to blood glucose levels, following bariatric surgery in a timely manner, could, in fact, reduce the incidence of adverse cardiovascular events.

A beneficial improvement of the cardiac structure and function could also be seen following the surgery, evidenced by the echocardiogram results done months after the study. It is safe to state that not only does bariatric surgery significantly reduce risk factors, but it also aids in sustaining the enhanced metabolism when compared to other alternative modalities of obese treatment, such as diet control. This reasoning could eventually assist all physicians in recommending gastric bypass surgeries to patients who suffer from these comorbidities, knowing that the benefits outweigh the risks, thus enhancing the healthspan and longevity of a patient with such a profile. There are still gaps to fill on debatable issues in this premise, to name one, such as the hypertension remission after bariatric surgery; however, more and more studies are emerging to pinpoint decisive conclusions to this ailment since it has a wide range of symptoms.
